# The Relationship of Peripheral Glycoprotein Nonmetastatic Melanoma Protein B and Parkinson′s Disease: Insights From a Mendelian Randomization Study

**DOI:** 10.1155/ijog/2340381

**Published:** 2026-10-09

**Authors:** Yaoyi Zhang, En Huang, Nan Lin

**Affiliations:** ^1^ Fujian Key Laboratory of Vascular Aging Department of Geriatrics Fujian Institute of Geriatrics Fujian Clinical Research Center for Senile Vascular Aging and Brain Aging Fujian Medical University Union Hospital Fuzhou Fujian China fjxiehe.com; ^2^ Key Laboratory of Brain Aging and Neurodegenerative Diseases of Fujian Province School of Basic Medical Sciences Fujian Medical University Fuzhou Fujian China fjmu.edu.cn; ^3^ Institute of Medical Sciences Fujian Medical University Union Hospital Fuzhou Fujian China fjxiehe.com

**Keywords:** expression quantitative trait loci (eQTL), glycoprotein nonmetastatic melanoma protein B (GPNMB), Mendelian randomization analysis, Parkinson′s disease, protein quantitative trait loci (pQTL)

## Abstract

**Background:**

Genome‐wide association studies (GWASs) have revealed that glycoprotein nonmetastatic melanoma Protein B (GPNMB) was associated with Parkinson′s disease (PD). GPNMB has been found in plasma and cerebrospinal fluid, and its levels and the variation of single nucleotide polymorphisms (SNPs) have been linked to disease severity in PD patients. However, whether the altered expression of GPNMB is the cause or the result cannot be identified in the patient samples.

**Methods:**

This study applied a Mendelian randomization analysis to explore the causal relationship between GPNMB and PD. The blood GPNMB mRNA level from the expression quantitative trait loci (eQTL) dataset and the plasma GPNMB protein level from the protein quantitative trait loci (pQTL) acted as the exposure. PD′s GWAS dataset from the International Parkinson′s Disease Genomics Consortium including 33674 PD cases and 449056 normal controls was applied as the outcome.

**Results:**

MR analysis with the inverse‐variant weighted method (IVW, OR = 1.416, *p* = 1.39*e* − 06) and the weighted median methods (WM, OR = 1.430, *p* = 4.47*e* − 07) showed that the plasma GPNMB protein expression was linked to a higher risk of PD. We also discovered a significant association between the blood mRNA level of GPNMB and PD risk. The IVW (OR = 1.243, *p* = 3.64*e* − 09) and WM (OR = 1.237, *p* = 5.11*e* − 08) methods share similar results. Secondary MR analysis further reveals causality between GPNMB pQTL/eQTL and brain volume traits.

**Conclusions:**

The study demonstrated a significant association between plasma GPNMB protein (pQTL) and mRNA level (eQTL) with PD. Further secondary MR analysis revealed causality between GPNMB pQTL/eQTL and brain volume traits.

## 1. Introduction

Parkinson′s disease (PD) is the second most common neurodegenerative disorder, and the global prevalence is about 2%–3% of the aging (≥ 65 years old) population [1]. Its clinical features mainly include motor symptoms such as bradykinesia and resting tremors and nonmotor features such as cognitive impairment and sleep disorders [1]. PD is caused by a combination of environmental factors and genetic variants. By now, some classic PD‐associated loci, like SNCA, PARK7, and PINK1, have been well characterized [2]. Genome‐wide association studies (GWASs) have revealed numerous risky genes associated with PD, including glycoprotein nonmetastatic melanoma protein B (GPNMB) [3–6]. GPNMB is a transmembrane glycoprotein and was first found to be expressed in melanoma cell lines including macrophages and microglia [7]. Recent studies showed that GPNMB also was involved in inflammation response in the brain and the periphery. GPNMB may be a crucial biomarker in the cerebrospinal fluid (CSF) of PD [8] and is highly expressed in the plasma of PD patients compared with normal people [8]. There was evidence suggesting that GPNMB had anti‐inflammatory and protective functions in some models of neurological diseases [9].

It has been reported that the two single nucleotide polymorphisms (SNPs) of GPNMB rs156429 and rs199347 were associated with the risk for PD [6, 10–12]. Moreover, GPNMB was found to be elevated in both plasma and CSF, associating with disease severity in PD with GPNMB SNP rs199347 [6]. However, whether the altered expression of GPNMB is the cause or the result cannot be identified in the patient samples. This study applied a Mendelian randomization (MR) analysis to assess the causal relationship between GPNMB and PD. MR employed genetic variants as the exposure, and the inheritance of the genetic variants is independent and randomly assorted. This random assortment of genetic variants mimics randomization used in clinical trials. Thus, it can overcome some limitations in observational studies with many potential biases, confounding, and reverse causation. This makes MR possible to ascertain the relatively robust causal effects of some risk on the outcomes or disease of interest [13].

## 2. Materials and Methods

### 2.1. GWAS Datasets

For the expression of GPNMB, we employed two GWAS datasets: One is the blood GPNMB mRNA level from the expression quantitative trait locus (eQTL) analysis of gene expression, which is derived from IEU open GWAS project (http://mrcieu.ac.uk) from 31,300 individuals of European descent (OpenGWAS ID: eqtl‐a‐ENSG00000136235). An eQTL is a locus that explains a fraction of the genetic variance with gene expression levels and provides a means for detecting transcriptional regulatory relationships at a genome‐wide scale [14]. The second is the plasma GPNMB protein level from pQTL analysis in 35,559 individuals of Icelanders from European Variation Archive (Accession Number PRJEB15197) [15]. These two datasets (eQTL/pQTL) acted as the exposure of MR analysis. Two GWAS datasets of PD were applied as the outcome of this MR analysis. One was from the PD dataset from the International Parkinson′s Disease Genomics Consortium (http://pdgenetics.org), which includes 33,674 PD cases and 449,056 normal controls to identify risk loci associated with PD. Besides, the second dataset is a series of brain volumes that may relate to PD that come from the studies of brain imaging phenotypes in the UK Biobank [16]. The details of both GWAS were listed in Table S1.

### 2.2. Filter of Eligible Instrumental Variables (IVs)

MR is a genetic method that applies IVs for genetic variants associated with the exposure to make causal inferences about the exposure on the outcome [17]. Hence, screening out the eligible IVs is most important for the first step of MR. All IVs need to meet the three assumptions (Figure 1). The first is the correlation assumption, which has a strong positive correlation (generally, *p* value no more than 5*e* − 6) between exposure and its SNP. The second is the independence assumption, which needs to exclude confounding factors that the obtained SNPs are irrelevant to outcome and exposure; the third is the exclusivity assumption, in which the SNPs we choose related to exposure must be uncorrelated to outcome (normally retaining the SNPs filtered by no less than the *p* value threshold within the correlation assumption we choose). We usually filter the eligible SNP by the robust threshold associated with exposure (*p* < 5*e* − 8). Sometimes, we may only harvest a limited number of SNPs significantly and we will use a moderate threshold (like *p* < 5*e* − 7 or *p* < 5*e* − 6) to get more SNPs [18]. We clumped and discarded SNPs at linkage disequilibrium (LD)*r* ^2^ > 0.001within a 10‐megabase (Mb) window based on a European ancestry, and we judged IVs using an*F*‐statistic of no less than 10. Furthermore, we searched the PhenoScanner database [19] for each IV to see whether it was directly associated with the outcome, and the relevant SNP was removed. All instrumental variables were derived from cisexpression quantitative trait loci (cis‐eQTLs) and cisprotein quantitative trait loci (cis‐pQTLs) to enhance specificity. For GPNMB, cisvariants were defined as SNPs located within a ± 1 Mb genomic window around the transcription start site of the GPNMB gene. The details were listed in Tables S2 and S3.

**Figure 1:**
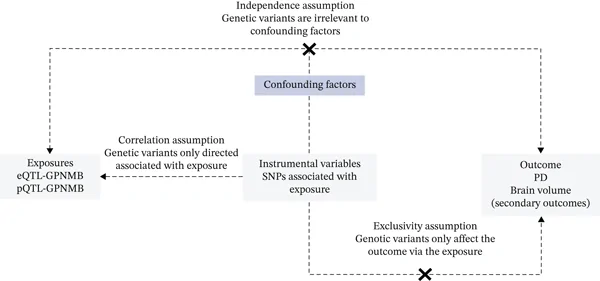
Flow diagram of the process for Mendelian randomization analysis in this study. eQTL, expression quantitative trait loci; GWAS, genome‐wide association studies; GPNMB, glycoprotein nonmetastatic melanoma protein B; pQTL, protein quantitative trait loci; SNP, single nucleotide polymorphisms.

### 2.3. Two‐Sample MR

The overall experimental design was shown in Figure 2 and the primary and secondary MR analysis is aimed at finding the causal effect of exposures on outcomes. Here, three MR methods were applied and included: inverse‐variance weighted (IVW) regression method, weighted median (WM) method, and MR–Egger regression method. The most important and efficient among these three methods was IVW, which could precisely weigh the genetic associations and return an unbiased estimate if the pleiotropy was null or balanced [20]. WM method was a robust method for heterogeneity; it estimated the causal effect as the median value of the genetic associations after ordering and weighing them by sample size; the median was less influenced by outlier results than the mean [21]. The MR–Egger regression method is mainly aimed at assessing the horizontal pleiotropy and provided a valid estimate for the pleiotropic effects not correlated with the strength of the genetic instruments; the intercept of MR–Egger represented the average horizontal pleiotropic effect across the exposure of genetic instruments [22]. Moreover, additional sensitivity analyses were further conducted to assess and evaluate potential associations [20]. The Cochran′s *Q* test, MR‐PRESSO, and MR–Egger intercept were applied to test the IVs heterogeneity, the outlier IVs, and the presence of directional pleiotropy, respectively [23]. After horizontal pleiotropy tests, the PhenoScanner database search was performed to ensure the valid MR results and exclude potential confounding factors. We performed MR analyses by TwoSampleMR and MR‐PRESSO packages in R Version 4.3.0.

**Figure 2:**
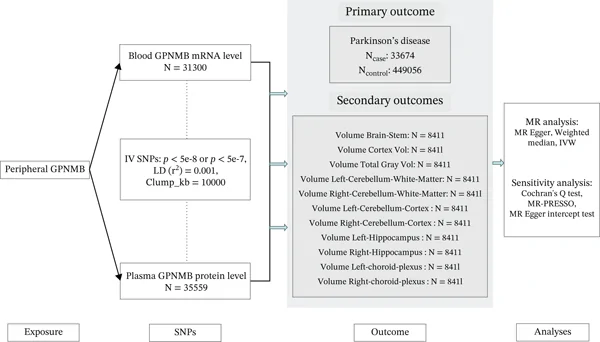
The overall experimental design of Mendelian randomization analysis in the present study.

## 3. Result

### 3.1. The Primary R Analyses Unveiled the Causal Effects Between pQTL/eQTL and PD

We applied pQTL/eQTL as the exposure to analyze the causal effect of the expressions of GPNMB in blood on PD as the outcome. It showed that the plasma GPNMB protein level (pQTL) was significantly associated with PD (Figure 3). The IVW (OR = 1.416, 95% CI: [1.230, 1.631], *p* = 1.39*e* − 06) showed that the plasma GPNMB protein expression was linked to a higher risk of PD, and WM (OR = 1.430, 95% CI: [1.244, 1.643], *p* = 4.47*e* − 07) had the similar result (Figure 3). Cochran *Q* test and MR–Egger intercept indicated no significant horizontal pleiotropy and heterogeneity, whereas the MR‐PRESSO showed the existence of pleiotropy, which may lead to the potential bias of the estimates. In this study, there was no clear evidence representing heterogeneity analyzed with Cochran′s *Q* and MR‐PRESSO tests. Taken together, although no outliers were identified, nominal between‐study heterogeneity may still exist according to MR‐PRESSO (Figure 4). We ought to compare them in another way, like the WM method and MR–Egger method, to further validate before firm conclusions can be drawn.

**Figure 3:**
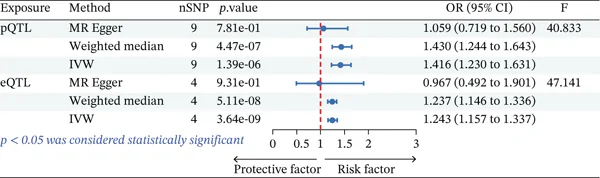
Forest plot for the causal effect of plasma GPNMB pQTL/eQTL on PD risk. pQTL/eQTL served as the exposure, and PD served as the outcome.

**Figure 4:**
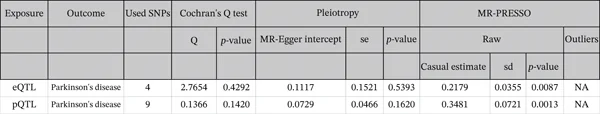
Heterogeneity and pleiotropy tests for the associations of plasma GPNMB eQTL/pQTL with PD.

On the other hand, in the blood GPNMB mRNA level, we also discovered that there was a significant association between the blood mRNA level of GPNMB and PD risk. The IVW (OR = 1.243, 95% CI = [1.157, 1.337], *p* = 3.64*e* − 09) and WM (OR = 1.237, 95% CI = [1.146, 1.336], *p* = 5.11*e* − 08) methods shared similar results from the serum GPNMB (Figure 3). The Cochran′s *Q* test and MR–Egger intercept indicated no horizontal pleiotropy and heterogeneity across studies, and MR‐PRESSO showed similar results as that of pQTL (Figure 4). Besides, we used *F*‐statistic to judge the whole IVs of pQTL and eQTL, and both passed the threshold.

### 3.2. Secondary MR Analysis Further Reveals Causality Between pQTL/eQTL and Brain Volume Traits

PD had some alteration in brain volume, and here, the brain volume served as the outcome for further investigating the causal effect among GPNMB pQTL/eQTL and some brain volume traits. When the GPNMB pQTL served as the exposure, we obtained a lot of significant associations between pQTL and brain volume (Figure 5). The plasma GPNMB protein level was significantly associated with increased volume of the brain‐stem (IVW: *β* = 0.086, 95% CI = [0.019, 0.153], *p* = 0.012), CSF (IVW: *β* = 0.098, 95% CI = [0.014, 0.182], *p* = 0.022), and right choroid plexus (IVW: *β* = 0.097, 95% CI = [0.022, 0.172], *p* = 0.011). Left choroid plexus was close to the threshold (IVW: *β* = 0.076, 95% CI = [−0.001, 0.153], *p* = 0.053). Oppositely, the blood GPNMB mRNA level was significantly associated with a decreased cortex volume (IVW: *β* = −0.053, 95% CI = [−0.101, −0.004], *p* = 0.033) (Figure 5). The Cochran′s *Q* test did not find heterogeneity in the association with the meaningful volume above (*p* > 0.05). Furthermore, MR‐PRESSO did not find heterogeneity, except in the volume of CSF and left choroid plexus (Figure 6).

**Figure 5:**
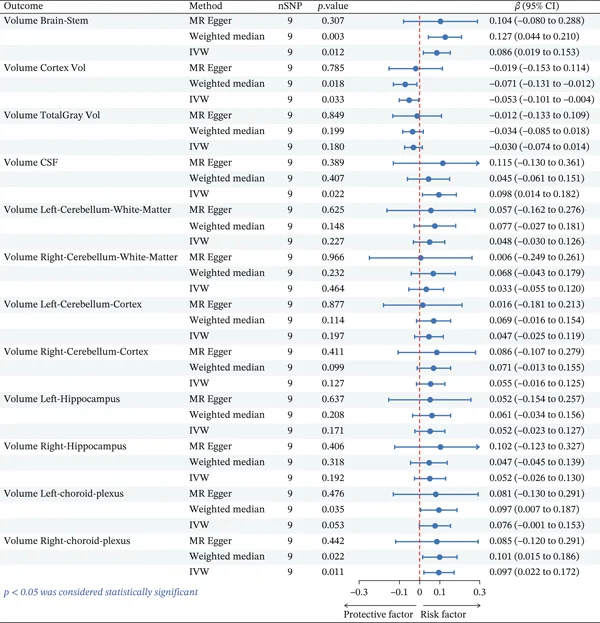
Forest plot for the causal effect of plasma GPNMB protein level (pQTL) on volume brain traits. pQTL as the exposure and brain volume traits as the outcome.

**Figure 6:**
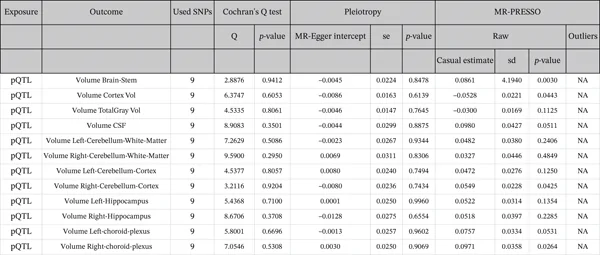
Heterogeneity and pleiotropy tests for plasma GPNMB protein level (pQTL) associations with PD and brain volume traits.

When the eQTL served as the exposure, a series of significant associations with brain volumes were also found (Figure 7). The blood GPNMB mRNA level was significantly associated with an increased volume of the brain stem (IVW: *β* = 0.060, 95% CI = [0.016–0.104], *p* = 0.008), right cerebellum white matter (IVW: *β* = 0.056, 95% CI = [0.003, 0.108], *p* = 0.037), left cerebellum cortex (IVW: *β* = 0.057, 95% CI = [0.010, 0.104], *p* = 0.018), right cerebellum cortex (IVW: *β* = 0.057, 95% CI = [0.002, 0.112], *p* = 0.041), left choroid plexus (IVW: *β* = 0.054, 95% CI = [0.003, 0.104], *p* = 0.037), and right choroid plexus (IVW: *β* = 0.064, 95% CI = [0.009, 0.119], *p* = 0.022). Conversely, the serum GPNMB protein level was significantly associated with decreased volume in the cortex (IVW: *β* = −0.043, 95% CI = [−0.075, −0.011], *p* = 0.009) (Figure 7). The Cochran′s *Q* test did not find heterogeneity in the association with the meaningful volume above (*p* > 0.05). However, the MR‐PRESSO did the potential bias to the estimates above in the brain stem, left choroid plexus, and cortex volume. The MR–Egger intercept did not find pleiotropy, except for the volume of right cerebellum white matter and the cortex (Figure 8).

**Figure 7:**
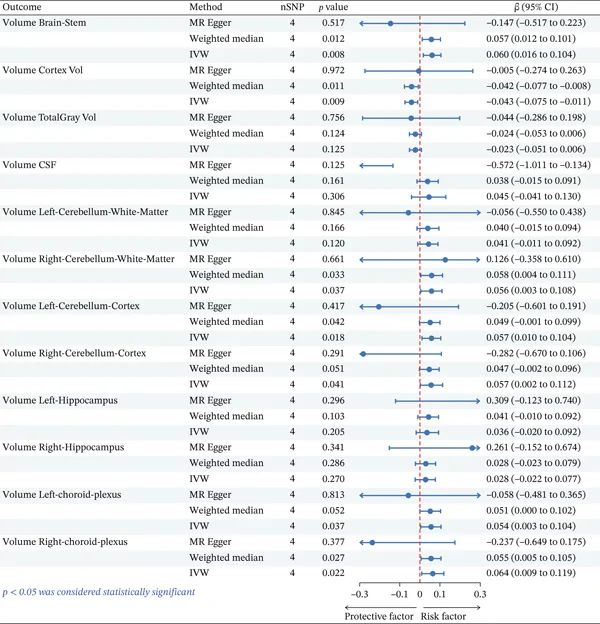
Forest plot for the causal effect of plasma GPNMB eQTL on volume brain traits. eQTL served as the exposure, and brain volume traits served as the outcome.

**Figure 8:**
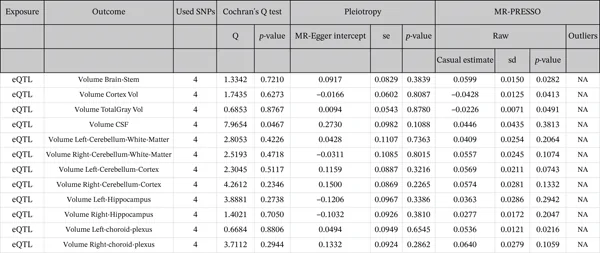
Heterogeneity and pleiotropy tests for plasma GPNMB mRNA level (eQTL) associations with PD and brain volume traits.

## 4. Discussion

It was reported that GPNMB SNPs were associated with the PD risk, and there is an altered protein level of GPNMB in plasma and CSF. However, whether the altered expression of GPNMB was the cause or the result could not be identified in the patient samples. This study systematically investigated the relationship between GPNMB and PD with MR analysis. The blood GPNMB mRNA level and the plasma GPNMB protein level served as the exposure showing a link to a higher risk of PD. PD had some alterations in brain volume, and the secondary MR analysis further reveals causality between GPNMB pQTL/eQTL and brain volume traits.

GPNMB is a Type‐I transmembrane glycoprotein that is first identified in osteoblasts [24]. Research indicated that GPNMB had anti‐inflammatory and neuroprotective functions [9, 25]. A recent study linked the GWAS‐derived CPNMB SNP rs199347 to PD risk via colocalization analyses of eQTL [6]. It also showed that GPNMB was elevated in PD plasma and associated with disease severity. GPNMB interacted with alpha‐synuclein (aSyn) and internalized aSyn, and GPNMB knockout led to dysregulated internalization of aSyn fibrils for degradation and progressed aSyn pathology [6]. Other several studies supported the relationship between GPNMB rs156429 and PD [10–12]. However, GWAS and QTL cannot approve the cause or result relationship between GPNMB and PD. MR analysis can be applied to ascertain the causal relationship between genes and neurological disorders including PD [13]. MR studies were conducted by integrating GWAS and eQTL data in dopaminergic neurons and nominated some risk genes that may have a causal role in PD, illuminating the potential therapeutic targets of PD [26–27]. Studies used the summary‐based MR (SMR) analysis to indicate that the expression and methylation of the endosomal membrane trafficking gene can predict PD risk [28–29].

In this MR analysis, the most fundamental and important was that the GPNMB pQTL/eQTL must be a valid IV [30]. In the present study, we have selected the IVs from large GWAS datasets with more than 30,000 individuals, and the SNPs passed the strong GWAS threshold at *p* < 5*e* − 08 and/or *F*‐statistic > 10. The horizontal pleiotropy tests and the PhenoScanner database search were performed, and all processes were to ensure that our MR results were valid. When the findings from IVW and WM methods were statistically significant, the MR–Egger intercept test was conducted to detect potential directional pleiotropy and showed that there were no horizontal pleiotropy and heterogeneity across studies (*p* > 0.05). Directional pleiotropy might arise when the genetic instruments were associated with the outcome through independent pathways of the exposure. This could lead to overestimation or underestimation of the true causal effect in the standard MR methods like IVW. The MR–Egger method was potentially able to provide consistent causal estimates in the presence of pleiotropy by regression of the estimates on their association with the outcome. However, the power of MR–Egger was lower compared with other methods. Additionally, MR–Egger was more prone to bias from horizontal pleiotropy compared with IVW and WM. Taken together, although IVW and WM methods detected significant associations, directional pleiotropy cannot be ruled out based on the MR–Egger results. This left some uncertainty regarding the magnitude and, possibly, the direction of the true causal effect. Further studies were warranted to validate the findings using alternative genetic instruments or approaches.

Widespread or local changes in brain volume were observed in a range of neurological disorders via clinical imaging techniques and especially related to some pertinent aspects of the pathophysiology of neurodegenerative disorders, including Alzheimer′s and PD [31]. Here, our investigation further reveals the correlation between GPNMB pQTL/eQTL and brain volume traits with the secondary MR analysis, supporting the finding that GPNMB SNP are associated with altered brain volume traits and neurodegeneration including PD.

However, there are limitations to consider. First, as the GWAS data were derived primarily from individuals of European ancestry, the generalizability of these results to non‐European groups may be constrained. Second, MR analyses cannot capture nongenetic variance. Third, colocalization analysis was not performed, which could provide valuable supportive evidence by quantifying the probability that the same underlying genetic variant influences both the exposure (GPNMB levels) and the outcome (PD or brain volumes). Therefore, the results should be interpreted with caution. Future research integrating other biomarker data will be crucial to further elucidate these causal relationships.

## 5. Conclusion

This study systematically investigates the relationship between peripheral GPNMB (pQTL and eQTL) and PD, using MR analysis. The study demonstrated a significant association between plasma GPNMB protein (pQTL) and mRNA level (eQTL) with PD. Further secondary MR analysis revealed the correlation between GPNMB pQTL/eQTL and brain volume traits. Future studies will investigate whether GPNMB can act as a biomarker and therapeutic target in PD.

## Author Contributions

Yaoyi Zhang and Nan Lin designed experiments and data analyses; En Huang provided technical support; Yaoyi Zhang and Nan Lin wrote the manuscript; and Nan Lin supervised the project. All authors have reviewed the manuscript.

## Funding

This study is funded by the National Natural Science Foundation of China (10.13039/501100001809, 82071562) and the Joint Funds for the Innovation of Science and Technology, Fujian Province (2023Y9154).

## Ethics Statement

The authors have nothing to report.

## Consent

The authors have nothing to report.

## Conflicts of Interest

The authors declare no conflicts of interest.

## Supporting information


**Supporting Information** Additional supporting information can be found online in the Supporting Information section. Table S1: Detailed information of the GWAS datasets. Specifically, the dataset of pQTL was decoded through the original data to obtain the cis‐pQTL (*r* ^2^ = 0.001, clump 1 Mb, and *p* < 5*e* − 8), and then we net screen out the pQTL data of the GPNMB for the MR analysis. Table S2: Detailed information of the screened SNPs in the pQTL dataset in the Mendelian randomization analysis in this study. Table S3: Detailed information of the screened SNPs in the eQTL dataset in the Mendelian randomization analysis in this study.

## Data Availability

The data that support the findings of this study are available from the corresponding author upon reasonable request.

## References

[1] Poewe W. , Seppi K. , Tanner C. M. , Halliday G. M. , Brundin P. , Volkmann J. , Schrag A. E. , and Lang A. E. , Parkinson Disease, Nature Reviews Disease Primers. (2017) 3, no. 1, 17013, 10.1038/nrdp.2017.13. 28332488

[2] Vazquez-Velez G. E. and Zoghbi H. Y. , Parkinson′s Disease Genetics and Pathophysiology, Annual Review of Neuroscience. (2021) 44, no. 1, 87–108, 10.1146/annurev-neuro-100720-034518. 34236893

[3] Gu X. J. , Su W. M. , Dou M. , Jiang Z. , Duan Q. Q. , Yin K. F. , Cao B. , Wang Y. , Li G. B. , and Chen Y. P. , Expanding Causal Genes for Parkinson′s Disease via Multi-Omics Analysis, NPJ Parkinson′s Disease. (2023) 9, no. 1, 10.1038/s41531-023-00591-0, 37865667. PMC10590374 37865667

[4] International Parkinson′s Disease Genomics Consortium , A Two-Stage Meta-Analysis Identifies Several New Loci for Parkinson′s Disease, PLoS Genetics. (2011) 7, no. 6, e1002142, 10.1371/journal.pgen.1002142, 21738488. PMC3128098 21738488

[5] Smajić S. , Prada-Medina C. A. , Landoulsi Z. , Ghelfi J. , Delcambre S. , Dietrich C. , Jarazo J. , Henck J. , Balachandran S. , Pachchek S. , and Morris C. M. , Single-cell Sequencing of Human Midbrain Reveals Glial Activation and a Parkinson-Specific Neuronal State, Brain. (2022) 145, no. 3, 964–978, 10.1093/brain/awab446, 34919646. PMC9050543 34919646

[6] Diaz-Ortiz M. E. , Seo Y. , Posavi M. , Carceles Cordon M. , Clark E. , Jain N. , Charan R. , Gallagher M. D. , Unger T. L. , Amari N. , Skrinak R. T. , Davila-Rivera R. , Brody E. M. , Han N. , Zack R. , van Deerlin V. M. , Tropea T. F. , Luk K. C. , Lee E. B. , Weintraub D. , and Chen-Plotkin A. S. , GPNMB Confers Risk for Parkinson′s Disease Through Interaction With *α*-synuclein, Science. (2022) 377, no. 6608, eabk0637, 10.1126/science.abk0637, 35981040. PMC9870036 35981040

[7] Saade M. , Araujo de Souza G. , Scavone C. , and Kinoshita P. F. , The Role of GPNMB in Inflammation, Frontiers in Immunology. (2021) 12, 674739, 10.3389/fimmu.2021.674739, 34054862. PMC8149902 34054862

[8] Hansson O. , Kumar A. , Janelidze S. , Stomrud E. , Insel P. S. , Blennow K. , Zetterberg H. , Fauman E. , Hedman Å. K. , Nagle M. W. , Whelan C. D. , Baird D. , Mälarstig A. , and Mattsson-Carlgren N. , The Genetic Regulation of Protein Expression in Cerebrospinal Fluid, EMBO Molecular Medicine. (2023) 15, no. 1, e16359, 10.15252/emmm.202216359, 36504281. PMC9832827 36504281

[9] Budge K. M. , Neal M. L. , Richardson J. R. , and Safadi F. F. , Glycoprotein NMB: An Emerging Role in Neurodegenerative Disease, Molecular Neurobiology. (2018) 55, no. 6, 5167–5176, 10.1007/s12035-017-0707-z, 28856541. 28856541

[10] Liu J. , Li G. , He Y. , He G. , Zhang P. , Shen X. , Zhang W. , Chen S. , Cui S. , and Tan Y. , The Association Analysis of GPNMB rs156429 With Clinical Manifestations in Chinese Population With Parkinson′s Disease, Frontiers in Genetics. (2020) 11, 10.3389/fgene.2020.00952, 32983228. PMC7492656 32983228

[11] Murthy M. N. , Blauwendraat C. , Ukbec G. S. , Ipdgc H. J. et al., Increased Brain Expression of GPNMB Is Associated With Genome Wide Significant Risk for Parkinson′s Disease on Chromosome 7p15.3, Neurogenetics. (2017) 18, no. 3, 121–133, 10.1007/s10048-017-0514-8, 28391543. PMC5522530 28391543

[12] Pihlstrøm L. , Axelsson G. , Bjørnarå K. A. , Dizdar N. , Fardell C. , Forsgren L. , Holmberg B. , Larsen J. P. , Linder J. , Nissbrandt H. , and Tysnes O. B. , Supportive Evidence for 11 Loci From Genome-Wide Association Studies in Parkinson′s Disease, Neurobiology of Aging. (2013) 34, no. 6, 1708.e7, 10.1016/j.neurobiolaging.2012.10.019. 23153929

[13] Allman P. H. , Aban I. B. , Tiwari H. K. , and Cutter G. R. , An Introduction to Mendelian Randomization With Applications in Neurology, Multiple Sclerosis and Related Disorders. (2018) 24, 72–78, 10.1016/j.msard.2018.06.017, 29960142. 29960142

[14] Nica A. C. and Dermitzakis E. T. , Expression Quantitative Trait Loci: Present and Future, Philosophical Transactions of the Royal Society B: Biological Sciences. (2013) 368, no. 1620, 20120362, 10.1098/rstb.2012.0362, 23650636. PMC3682727 23650636

[15] Ferkingstad E. , Sulem P. , Atlason B. A. , Sveinbjornsson G. , Magnusson M. I. , Styrmisdottir E. L. , Gunnarsdottir K. , Helgason A. , Oddsson A. , Halldorsson B. V. , Jensson B. O. , Zink F. , Halldorsson G. H. , Masson G. , Arnadottir G. A. , Katrinardottir H. , Juliusson K. , Magnusson M. K. , Magnusson O. T. , Fridriksdottir R. , Saevarsdottir S. , Gudjonsson S. A. , Stacey S. N. , Rognvaldsson S. , Eiriksdottir T. , Olafsdottir T. A. , Steinthorsdottir V. , Tragante V. , Ulfarsson M. O. , Stefansson H. , Jonsdottir I. , Holm H. , Rafnar T. , Melsted P. , Saemundsdottir J. , Norddahl G. L. , Lund S. H. , Gudbjartsson D. F. , Thorsteinsdottir U. , and Stefansson K. , Large-Scale Integration of the Plasma Proteome With Genetics and Disease, Nature Genetics. (2021) 53, no. 12, 1712–1721, 10.1038/s41588-021-00978-w. 34857953

[16] Elliott L. T. , Sharp K. , Alfaro-Almagro F. , Shi S. , Miller K. L. , Douaud G. , Marchini J. , and Smith S. M. , Genome-Wide Association Studies of Brain Imaging Phenotypes in UK Biobank, Nature. (2018) 562, no. 7726, 210–216, 10.1038/s41586-018-0571-7, 30305740. PMC6786974 30305740

[17] Lee K. and Lim C. Y. , Mendelian Randomization Analysis in Observational Epidemiology, Journal of Lipid and Atherosclerosis. (2019) 8, no. 2, 67–77, 10.12997/jla.2019.8.2.67, 32821701. PMC7379124 32821701

[18] Cai J. , He L. , Wang H. , Rong X. , Chen M. , Shen Q. , Li X. , Li M. , and Peng Y. , Genetic Liability for Prescription Opioid Use and Risk of Cardiovascular Diseases: A Multivariable Mendelian Randomization Study, Addiction. (2022) 117, no. 5, 1382–1391, 10.1111/add.15767, 34859517. 34859517

[19] Staley J. R. , Blackshaw J. , Kamat M. A. , Ellis S. , Surendran P. , Sun B. B. , Paul D. S. , Freitag D. , Burgess S. , Danesh J. , Young R. , and Butterworth A. S. , PhenoScanner: A Database of Human Genotype-Phenotype Associations, Bioinformatics. (2016) 32, no. 20, 3207–3209, 10.1093/bioinformatics/btw373, 27318201. PMC5048068 27318201

[20] Burgess S. , Davey Smith G. , Davies N. M. , Dudbridge F. , Gill D. , Glymour M. M. , Hartwig F. P. , Holmes M. V. , Minelli C. , Relton C. L. , and Theodoratou E. , Guidelines for Performing Mendelian Randomization investigations, Wellcome Open Research. (2019) 4, 10.12688/wellcomeopenres.15555.1. PMC7384151 32760811

[21] Burgess S. , Foley C. N. , Allara E. , Staley J. R. , and Howson J. M. M. , A Robust and Efficient Method for Mendelian Randomization With Hundreds of Genetic Variants, Nature Communications. (2020) 11, no. 1, 10.1038/s41467-019-14156-4, 31953392. PMC6969055 31953392

[22] Bowden J. , Del Greco M. F. , Minelli C. , Davey Smith G. , Sheehan N. A. , and Thompson J. R. , Assessing the Suitability of Summary Data for Two-Sample Mendelian Randomization Analyses Using MR-Egger Regression: The Role of the I2 Statistic, International Journal of Epidemiology. (2016) 45, no. 6, 1961–1974, 10.1093/ije/dyw220, 27616674. PMC5446088 27616674

[23] Verbanck M. , Chen C. Y. , Neale B. , and Do R. , Detection of Widespread Horizontal Pleiotropy in Causal Relationships Inferred From Mendelian Randomization Between Complex Traits and Diseases, Nature Genetics. (2018) 50, no. 5, 693–698, 10.1038/s41588-018-0099-7, 29686387. PMC6083837 29686387

[24] Safadi F. F. , Xu J. , Smock S. L. , Rico M. C. , Owen T. A. , and Popoff S. N. , Cloning and Characterization of Osteoactivin, a Novel cDNA Expressed in Osteoblasts, Journal of Cellular Biochemistry. (2001) 84, no. 1, 12–26, 10.1002/jcb.1259, 11746512. 11746512

[25] Yu B. , Sondag G. R. , Malcuit C. , Kim M. H. , and Safadi F. F. , Macrophage-Associated Osteoactivin/GPNMB Mediates Mesenchymal Stem Cell Survival, Proliferation, and Migration Via a CD44-Dependent Mechanism, Journal of Cellular Biochemistry. (2016) 117, no. 7, 1511–1521, 10.1002/jcb.25394, 26442636. 26442636

[26] Dang X. , Zhang Z. , and Luo X. J. , Mendelian Randomization Study Using Dopaminergic Neuron-Specific eQTL Nominates Potential Causal Genes for Parkinson′s Disease, Movement Disorders. (2022) 37, no. 12, 2451–2456, 10.1002/mds.29239, 36177513. 36177513

[27] Cui X. , Xu C. , Zhang L. , and Wang Y. , Identification of Parkinson′s Disease-Causing Genes via Omics Data, Frontiers in Genetics. (2021) 12, 712164, 10.3389/fgene.2021.712164, 34394198. PMC8355633 34394198

[28] Storm C. S. , Kia D. A. , Almramhi M. , and Wood N. W. , Using Mendelian Randomization to Understand and Develop Treatments for Neurodegenerative Disease, Brain Communications. (2020) 2, no. 1, fcaa031, 10.1093/braincomms/fcaa031, 32954289. PMC7425289 32954289

[29] Bandres-Ciga S. , Saez-Atienzar S. , Bonet-Ponce L. , Billingsley K. , Vitale D. , Blauwendraat C. , Gibbs J. R. , Pihlstrøm L. , Gan-Or Z. , The International Parkinson′s Disease Genomics Consortium (IPDGC) , Cookson M. R. , Nalls M. A. , and Singleton A. B. , The Endocytic Membrane Trafficking Pathway Plays a Major Role in the Risk of Parkinson′s Disease, Movement Disorders. (2019) 34, no. 4, 460–468, 10.1002/mds.27614, 30675927. PMC6467788 30675927

[30] Hemani G. , Zheng J. , Elsworth B. , Wade K. H. , Haberland V. , Baird D. , Laurin C. , Burgess S. , Bowden J. , Langdon R. , Tan V. Y. , Yarmolinsky J. , Shihab H. A. , Timpson N. J. , Evans D. M. , Relton C. , Martin R. M. , Davey Smith G. , Gaunt T. R. , and Haycock P. C. , The MR-Base Platform Supports Systematic Causal Inference Across the Human Phenome, Elife. (2018) 7, 10.7554/eLife.34408. PMC5976434 29846171

[31] Bozzali M. , Cercignani M. , and Caltagirone C. , Brain Volumetrics to Investigate Aging and the Principal Forms of Degenerative Cognitive Decline: A Brief Review, Magnetic Resonance Imaging. (2008) 26, no. 7, 1065–1070, 10.1016/j.mri.2008.01.044, 18436405. 18436405

