# Supplementary material for: The Relationship of Peripheral Glycoprotein Nonmetastatic Melanoma Protein B and Parkinson′s Disease: Insights From a Mendelian Randomization Study
Source: Int J Genomics. 2026 Oct 9;2026:2340381. doi: 10.1155/ijog/2340381 (PMC13651710; doi:10.1155/ijog/2340381)
Supplement: Supplementary file 1 [file IJOG-2026-2340381-s001.docx]

**Supplementary materials**

**Supplementary table 1.** Detailed information of the GWAS datasets. Especially, the dataset of pQTL was decoded through the original data to obtain the cis-pQTL (r2=0.001, clump1MB, and p<5e-8), and then we net screen out the pQTL data of the GPNMB for the MR analysis.

**Supplementary table 2.** Detailed information of the screened SNPs in the pQTL dataset in the Mendelian randomization analysis in this study.

**Supplementary table 3.** Detailed information of the screened SNPs in the eQTL dataset in the Mendelian randomization analysis in this study.
